# Repeat Instability in the Fragile X-Related Disorders: Lessons from a Mouse Model

**DOI:** 10.3390/brainsci9030052

**Published:** 2019-03-01

**Authors:** Xiaonan Zhao, Inbal Gazy, Bruce Hayward, Elizabeth Pintado, Ye Hyun Hwang, Flora Tassone, Karen Usdin

**Affiliations:** 1 Section on Gene Structure and Disease, Laboratory of Cell and Molecular Biology, National Institute of Diabetes, Digestive and Kidney Diseases, National Institutes of Health, Bethesda, MD 20892, USA; xiaonan.zhao@nih.gov (X.Z.); inbal.gazy@nih.gov (I.G.); bruce.hayward@nih.gov (B.H.); 2 Department of Medical Biochemistry and Molecular Biology, School of Medicine, University Hospital Virgen Macarena, University of Seville, 41009 Seville, Spain; elizabet@us.es; 3 Department of Biochemistry and Molecular Medicine and MIND Institute, UC Davis Medical Center, Sacramento, CA 95817, USA; yehhwang@ucdavis.edu (Y.H.H.); ftassone@ucdavis.edu (F.T.)

**Keywords:** CGG Repeat Expansion Disease, DNA instability, expansion, contraction, mismatch repair (MMR), base excision repair (BER), transcription coupled repair (TCR), double-strand break repair (DSBR), Non-homologous end-joining (NHEJ), mosaicism

## Abstract

The fragile X-related disorders (FXDs) are a group of clinical conditions that result primarily from an unusual mutation, the expansion of a CGG-repeat tract in exon 1 of the *FMR1* gene. Mouse models are proving useful for understanding many aspects of disease pathology in these disorders. There is also reason to think that such models may be useful for understanding the molecular basis of the unusual mutation responsible for these disorders. This review will discuss what has been learnt to date about mechanisms of repeat instability from a knock-in FXD mouse model and what the implications of these findings may be for humans carrying expansion-prone *FMR1* alleles.

## 1. Introduction

The fragile X-related disorders (FXDs) are X-linked disorders that include a form of ovarian dysfunction known as fragile X-associated primary ovarian insufficiency (FXPOI; MIM# 311360), a neurodegenerative condition, fragile X-associated tremor/ataxia syndrome (FXTAS; MIM# 300623) and fragile X syndrome (FXS; MIM# 300624), a major cause of intellectual disability and autism [1]. FXPOI and FXTAS are seen in carriers of *FMR1* premutation (PM) alleles, alleles that have a tandem array of 55–200 CGG repeats in exon 1. Most cases of FXS are seen in carriers of *FMR1* full mutation (FM) alleles, alleles that have >200 repeats, with a minority of individuals having deletions or point mutations that affect the levels or functionality of FMRP, the *FMR1* gene product, an important regulator of translation in the brain. The difference in the clinical consequences of the inheritance of a PM versus a FM allele results from the paradoxical effects of the repeat on *FMR1* expression. Most FM alleles are epigenetically silenced, resulting in the absence of FMRP. In contrast PM alleles are transcriptionally active and can have transcript levels anywhere between 2 and 8 times the levels of normal alleles [2]. Both FM and PM carriers show wide variability in their clinical presentation and both FXTAS and FXPOI show incomplete penetrance suggesting the contribution of other genetic factors to disease severity.

The CGG-repeat tract is unstable and is prone to expand and contract in a manner dependent on repeat number and the number of AGG interruptions present at the 5’ end of the tract [3,4]. Instability occurring in somatic cells can lead to repeat size mosaicism. In fact, it has been estimated that >40% of individuals with alleles >55 repeats are mosaic [5]. Mosaicism for both PM and FM alleles results in some individuals showing symptoms characteristic of both PM and FM alleles [6,7]. The mechanism(s) responsible for the repeat instability is largely unknown. While some instability has been reported in various human cells in culture [8,9,10], most studies of the instability mechanisms have used mouse models [11,12,13,14]. Mouse models offer a number of clear advantages over some of these cell-based systems, including the high frequency of both expansions and contractions and the ability to examine instability in different biologically relevant organs at various stages of development. In addition, since the size of the original allele can be readily established based on the allele size at birth, alleles arising from expansion can be clearly distinguished from those arising by contraction. Of the various mouse models for the FXDs that have been generated, most work on repeat instability has made use of a model in which the short CGG-repeat tract present in the endogenous mouse *FMR1* gene was replaced with ~130 CGG-repeats [14]. In this review we will address what we have learnt to date about repeat instability from this mouse model, as well as from other model systems and other related Repeat Expansion Disorders. We will also discuss some of the implications of this information for diagnosis and disease risk assessment in humans.

## 2. Instability in Humans and Mice May Share a Common Molecular Basis

There are a number of reasons to think that instability in mice and humans share common mechanisms. Firstly, while both expansions and contractions are seen, in both species expansions predominate over contractions, at least in the PM range. Secondly, in both humans and mice expansion events require transcription or the presence of the PM allele in a region of open chromatin [15,16]. In addition, both mice and humans show a maternal age effect for expansion risk [3,17], suggesting that expansion occurs in the oocyte in both species. While some postnatal oogenesis has been observed in mice whose existing oocytes have been ablated [18], the contribution of a dividing oocyte stem cell population to postnatal oogenesis and the pool of viable oocytes that can be fertilized in normal mammals is still controversial. Thus, since oocytes do not divide, a maternal age effect for expansions is generally considered to reflect events that occur in the absence of cell division. This suggests that the underlying mechanism in both species involves aberrant DNA repair and/or recombination, rather than a problem with chromosomal replication. Finally, many of the same genetic factors that affect expansion risk in the FXD mouse are known to modulate expansion risk in other human Repeat Expansion Diseases [19,20,21]. Since current evidence supports a common mutational mechanism for all of these diseases, this suggests that the FXD mouse may accurately recapitulate at least some aspects of repeat expansion in the FXDs.

Whilst most intergenerational expansions in mice are relatively small, large expansions characterize the PM to FM transition on maternal transmission in humans. However, there is no clear evidence that the PM to FM transition occurs in a single step in women and, in principle, it is possible to generate an equivalent increase in repeat number by a series of small expansions occurring over the decades that the human oocyte spends in dictyate arrest prior to fertilization [17]. Furthermore, while small expansions predominate in mice, larger intergenerational expansions are seen albeit at a lower frequency [17]. These larger expansions are sensitive to the same genetic factors that affect small expansions and thus likely arise from the same basic mechanism [22,23]. While much less is known about contractions, work in both mice and humans suggest that the underlying mechanism is likely different from the mechanism that gives rise to expansions [4,24,25]. 

Although available evidence suggests that mice may be useful for understanding the instability of human PM alleles, mouse *Fmr1* alleles with repeat numbers in the FM range do not undergo repeat-mediated epigenetic silencing as do FM alleles in humans. However, there is reason to think that X chromosome inactivation in female mice can provide a window into the factors associated with the instability of silenced alleles [16].

## 3. Different Cell Types Show Different Propensities to Expand in Mice

**Figure 1:**
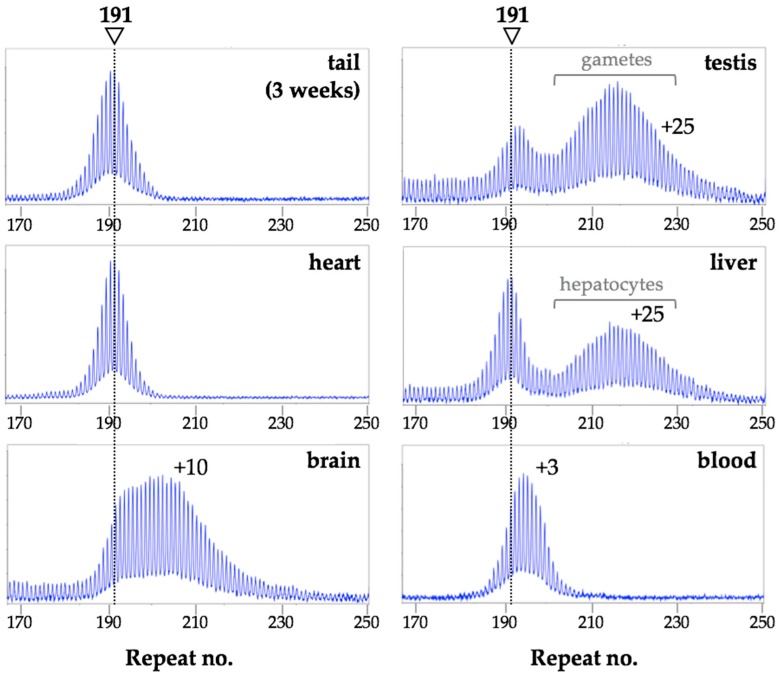
Different mouse organs show different propensities to expand. Repeat PCR was carried out on DNA extracted from different organs of a male mouse with 191 inherited repeats and analyzed as previously described [26]. The tail DNA sample was taken at 3 weeks, the remaining samples at 8 months of age. The arrowhead and dotted lines indicate the repeat number in the original inherited allele as assessed from tail DNA taken at 3 weeks of age. The numbers within each panel indicate the number of repeats added.

**Figure 2:**
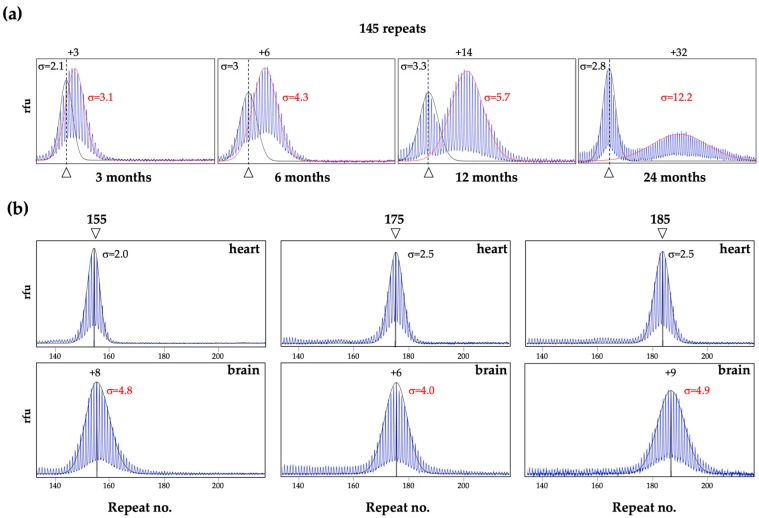
Change in the PM repeat PCR profiles and σ with age and extent of expansion. The PCR profiles and σs were generated from male mice as previously described [26,27]. (**a**) Liver PCR profiles of mice of different ages, all with an original allele of ~145 repeats. The arrowhead on the bottom of each panel and the dotted lines indicate the original inherited allele as assessed from tail DNA taken at 3 weeks of age. The numbers above the panels indicate the number of repeats added to the expanded allele. The σ of the stable allele and the expanded allele are shown in black and red, respectively. The examples shown in this panel are derived from previously published work [27]; (**b**) PCR profiles and corresponding σ for alleles in hearts and brains of 1-year old (155 repeats) and 6-month old (175 and 185 repeats) mice. The number associated with each arrowhead represents the number of repeats in the indicated allele. For the hearts, this number corresponds to the original inherited allele. For the brains, the repeat size reflects a gain of 6–9 repeats from the original allele.

As can be seen in Figure 1, in the FXD mice some organs, like heart, show very little post-natal expansion, as evidenced by the fact that the repeat PCR profile of this organ does not change with age and remains indistinguishable from the profile seen in the tail DNA taken at 3 weeks of age [26]. In contrast, most other organs show some expansion as evidenced by the presence of alleles larger than the heart allele (Figure 1) [17,26]. In organs like testes and liver, expansions are apparent as a shift from a unimodal repeat PCR profile as seen in the tail DNA, to one that is more bimodal (Figure 1 and Figure 2a). In young animals that have not yet accumulated many expansions, the second peak can appear more like a “shoulder” rather than a distinct peak, as in the example of the liver of a 3-month old mouse shown in Figure 2a. However, over time, as expansions continue to accumulate, expanding alleles diverge further from the original allele resulting in 2 clearly distinct allele peaks (Figure 1 and Figure 2a). This bimodal peak distribution reflects the fact that, in some organs some cells are expansion-prone whilst others are not. 

For example, in testes expansion is confined to the spermatogonia or primary spermatocytes [17], with the shorter alleles in the testes profile in Figure 1 corresponding to alleles in the somatic cells and the longer alleles corresponding to alleles in the gametes. Similarly, in the liver, expansion is confined to hepatocytes [27], while in the brain expansion is more extensive in the striatum and basolateral amygdala than in the medial prefrontal cortex [26]. Unlike PM alleles in testes and liver, PM alleles in blood show relatively little expansion and what little expansion is seen has a unimodal distribution in males (Figure 1). This may reflect the fact that all white blood cells are equally prone to expansion. Why some cells are expansion-prone and others are not is not fully understood. It does not seem to be simply related to the amount of *Fmr1* transcription since tissues with similar levels of *Fmr1* mRNA can show very different propensities to expand [26]. Rather it may be related to the balance between the levels of expression of proteins involved in the generation of expansions and those involved in the pathway(s) that promotes contraction or error-free repair [26,28]. Computer simulations suggest that the expanded allele profile, even that seen in very expansion-prone tissue, is consistent with the addition of 1–2 repeats with each expansion event [29]. However, as will be discussed below, larger expansions and contractions also occur (Section 7).

As alleles expand, their PCR profile widens as differences in the timing and size of expansions in different cells transforms the original discrete allele into a more heterogenous mixture of allele sizes (Figure 2a). Thus, the broadness of the allele peak, as reflected in the standard deviation (σ), can be a sensitive metric of expansion [27,29]. In our experience, stable alleles, like those in heart, show a σ of ≤2.5, for a wide range of repeat sizes and mouse ages as illustrated in Figure 2b. In contrast, the σ of alleles in expansion-prone cells is >2.5 and increases as expansion increases as the animals age (Figure 2a,b). Overlapping allele peaks can result in an overestimation of the σ of the smaller allele and an underestimation of the larger one (Figure 2a). However, in unimodal PCR profiles or profiles with distinct peaks, an allele that has expanded has a larger σ than a stable allele of the same size (compare brain to heart in Figure 2b). 

It should be noted that while the repeat PCR profiles for alleles in the heart of WT mice shows no evidence of post-natal expansion, they differ from the heart profiles in mice with mutations that abolish expansions completely [30]. Specifically, the profile in WT animals has a normal distribution while the profile in mutant mice is not only sharper, but is also left-skewed [30]. This left skew likely reflects PCR stutter products, while the normal distribution of the WT heart profiles likely reflects some expansion that occurs in these animals during early embryonic development.

**Figure 3:**
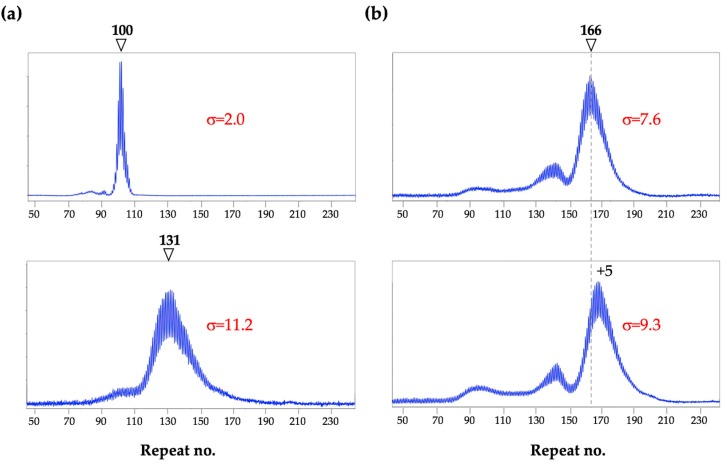
Blood repeat PCR profiles for 3 human male PM carriers. Repeat PCR analysis was carried out as described previously [31]. The number associated with each arrowhead represents the number of repeats in the indicated allele. (**a**) Profiles of two individuals showing a unimodal profile with a sharp peak (top) and a broad peak (bottom); (**b**) Profiles generated from the same individual using samples taken 4 years apart. The dotted line in these panels indicates the size of the major allele at the earlier timepoint. A shift corresponding to the gain of 5 CGG repeats is seen in the later sample. In each case the σ values are for the major allele peak.

*Implications for humans:* Analysis of the CGG repeat in the human *FMR1* gene is routinely performed using blood where, as in mice, a unimodal PCR profile is commonly seen in males. However, by analogy with what is seen in mice, a unimodal PCR profile may not mean that the allele is stable. As with mice, the σ of an allele profile likely reflects the extent of somatic expansion. This would be predicted to vary with total repeat number, the number of AGG interruptions and the effect of different genetic and/or environmental modifiers of expansion risk. As with mice (Figure 2a), age may also be a factor, at least for very unstable alleles. Figure 3a shows examples of unimodal repeat PCR profiles characteristic of stable (top panel) and unstable (bottom panel) alleles. 

As illustrated in Figure 3b, multiple peaks are also sometimes seen in males. These alleles may arise by contraction of larger alleles or from large expansions. As can be seen in the 2 different blood samples from the same individual taken 4 years apart (Figure 3b), these peaks can be broad, indicative of subsequent expansions. Notably, in this individual, both the repeat number and the σ were larger in the second sample taken 4 years later, consistent with an age effect. 

PM alleles are relatively stable in mouse blood compared to alleles from other cells. Thus, it is possible that any evidence of expansion in human blood, reflects the presence of even more extensive expansion in organs like brain or gonads. Different allele profiles in different tissues have been reported in some PM carriers [32,33,34,35,36,37,38,39] and 2 different studies support the idea that some men have larger expansions with broader σs in sperm relative to blood [40,41]. Differences between allele sizes in blood and in brain or gonads in expansion-prone individuals could lead to an underestimation of the intergenerational expansion risk. It may be that it also contributes to the apparent variability in the penetrance of FXTAS and FXPOI.

## 4. Expansions in Females Only Occurs on The Active X Chromosome

While in male mice a bimodal repeat PCR profile is seen in organs that have cell types with different expansion rates, most female mice show a bimodal PCR profile for the PM allele in all expansion-prone tissues [16]. This is a consequence of the fact that expansions in females are confined to alleles on the active X chromosome [16]. Thus, even in expansion-prone cell types, expansion only occurs in ~50% of cells in females with normal X chromosome inactivation (XCI). 

**Figure 4:**
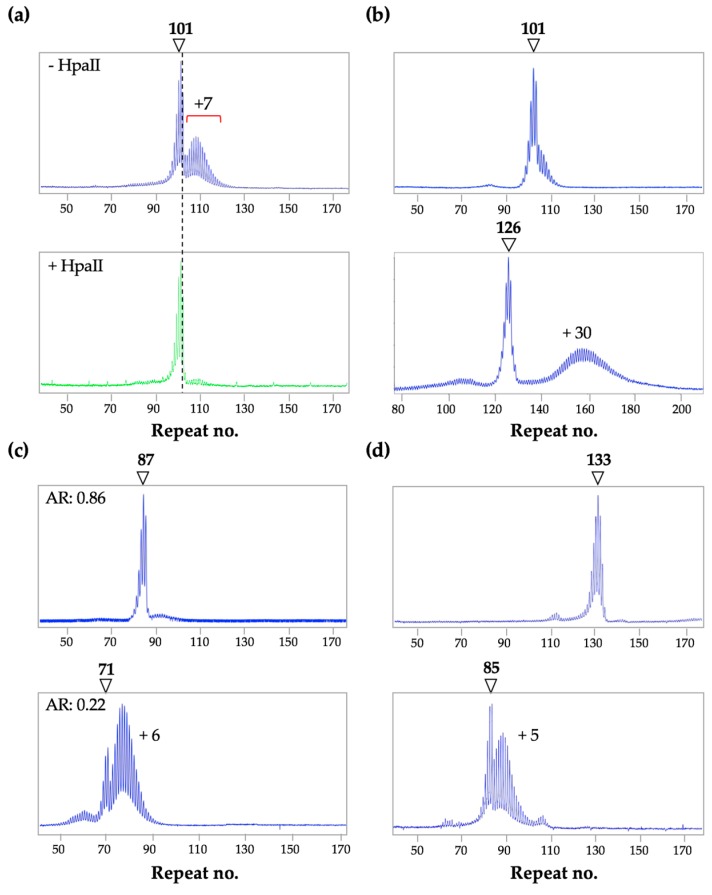
PM Repeat PCR profiles from the blood of human female PM carriers. The arrow in each instance indicates the stable allele with the indicated number of repeats. (**a**) Profiles for a female PM carrier without (top panel) and with (bottom panel) HpaII pre-digestion were generated as previously described [16]. The alleles on both the active and inactive X are shown in blue in the top panel and the alleles on the inactive X in green in the bottom panel; (**b**) Examples of very different bimodal PCR profiles; (**c**) Profiles for 2 women with different activation ratios (ARs); (**d**) Profiles of two females of similar ages and ARs, both with no AGG interruptions showing very different levels of somatic expansion.

*Implications for humans:* A bimodal PCR profile for the PM allele is also sometimes seen in human females (Figure 4). The larger alleles are lost from the repeat PCR profile when the DNA is digested prior to PCR with a methylation-sensitive enzyme that has one or more cleavage sites within the PCR amplicon. Such an enzyme cuts the *FMR1* allele on the active X chromosome, leaving the allele on the inactive X as the only template for PCR. Thus, in the example shown in Figure 4a, alleles on the active X have gained ~7 repeats relative to alleles on inactive X chromosomes (bottom panel). Notably, unlike the roughly normal distribution seen in the repeat PCR profiles of expanded alleles, the shape of the repeat PCR profile for alleles on the inactive X is asymmetric and closely resembles the shape of the PCR profile seen in mice with mutations that completely block expansions [30,42]. Even among women with a bimodal allele distribution, differences in the extent of instability can result in dramatic differences in their PCR profiles. Figure 4b illustrates the 2 extremes of the possible bimodal PCR profiles, with the woman in the top panel showing a very low level of somatic instability and the woman in the bottom panel showing unusually high levels. 

However, not all women show a bimodal profile for the PM allele. For example, as shown in the upper panel of Figure 4c, women with a high activation ratio (AR), that is, a high proportion of cells in which the active X chromosome carries the normal allele, would show a single sharp peak for the PM allele, with little, if any, evidence of a second peak, since no expansion would take place on the inactive X. In contrast, a woman with a low AR, would be more likely to have a profile with more expanded alleles than stable ones as shown in the bottom panel of Figure 4c. An even lower AR might result in unimodal PM profile with a large σ, as reported for a woman with an AR of 0.06 [43]. A single, sharp PM allele profile can also be seen even in the absence of skewed XCI (Figure 4d). Such alleles may result from the presence of AGG interruptions that reduce the expansion frequency, a genetic background that is not prone to expansion or, potentially, to an effect of age, with very young females being more likely to show such a profile. In the cases shown in Figure 4d, both women were of similar age, showed no skewing of XCI and had no AGG interruptions. Thus, the sharp and asymmetric profile seen for the 133 repeat allele may reflect genetic or environmental factors that reduce expansion risk as in mice with mutations that block expansions [24,30,42].

## 5. Expansion in the Male and Female Germline 

More expansions are seen in the oocytes of older female mice than younger ones [17]. This is consistent with some expansions occurring postnatally in non-dividing oocytes. In contrast, germ line expansions in male mice occurs in the spermatogonial stem cells (SSCs), cells that undergo multiple rounds of cell division [17]. Furthermore, male mice show a higher frequency of germ line expansions than females [17]. Studies of mice with mutations in different genes shows that the same genetic factors affect expansions in males and females and the same genetic factors also account for large and small expansions [24,25,28,30,42,44]. This would be consistent with single mechanism being responsible for all expansions in both males and females.

*Implications for humans:* Almost all expansions from a PM to a FM allele in humans occurs on maternal transmission. However, men with PM alleles transmit more expansions at least for smaller PM alleles [4,45] consistent with what is observed in mice. The fact that expansions in male and female mice share a dependence on a common set of genes would argue against a female-specific mechanism for the generation of FM alleles in humans. The FX repeat forms unusual intrastrand secondary structures [46,47,48,49,50], that are thought to make the repeat tract difficult to replicate [49,51,52]. This may result in pressure for larger alleles in dividing gametes to contract over time, as is seen in FX embryonic stem cells [53]. Germ cells in a 30-year old man will have undergone ~400 divisions, compared to 31 in a woman the same age [54,55]. However, by analogy with mice, most expansions in the female germline likely occur during post-natal life, well after cell division is complete. Thus, expanded alleles in female gametes face little pressure to contract, whilst male gametes are under continuous pressure to do so. This might explain why FMRP was detected in primordial germ cells of a 17-week old male FM fetus but not in those of a 13-week old fetus [56] and why older FM males only have PM alleles in their sperm [57,58]. It may also provide an explanation for why male PM carriers do not generally transmit FM alleles to their children. 

## 6. Genetic and Environmental Factors Affecting Instability

A number of genetic and environmental factors have been shown to impact expansion risk in mice. For example, an exogenous source of oxidative stress increases expansions [59]. Mutations in different DNA repair genes also affects the extent of expansion, with some mutations reducing expansions [24,25,28,30,42,44] and others increasing them [27,30,44,60]. For example, mutations in mismatch repair (MMR) proteins, including MSH2, MSH3, MSH6 and MLH3, either eliminate expansions altogether [24,30,42] or severely reduce their incidence [25]. Similarly, a single hypomorphic allele of Polβ, a DNA polymerase that plays an essential role in base excision repair (BER), is sufficient to significantly reduce the expansion frequency [28]. Thus, proteins from multiple DNA repair pathways that normally work to prevent mutations, interact in such a way so as to actually cause the repeat expansion mutation. Work in vitro has shown that the FX repeats form unusual DNA structures including hairpins that have a mixture of Watson-Crick and non-Watson-Crick base pairs [46,47,48,49,50,61,62]. Current thinking is that these structures are the substrates upon which this process acts but the sequence of events and all the factors involved are still not fully understood (see [23] for recent review). 

Mutations in other proteins, including two 5′-3′ exonucleases, EXO1 and FAN1, lead to an increase in expansions, suggesting that these proteins are protective [30,60]. Loss of EXO1 affects expansions in the germ line and in the small intestine but not in the brain [30]. In contrast, loss of FAN1 affects expansion in multiple organs including brain but does not affect the germ line expansion frequency [60]. ERCC6/CSB plays a paradoxical role in repeat expansion playing a minor role in promoting expansions in some instances [44] and protecting against them in others [63]. This paradoxical effect may reflect this protein’s ability to participate in multiple DNA repair pathways. Recently, DNA ligase IV (LIG4) has also been shown to protect against expansion [27]. Since LIG4 is essential for non-homologous end-joining (NHEJ), a form of double strand break (DSB) repair, it suggests that the expansion process competes with the NHEJ pathway for a common substrate. This supports the idea that expansion proceeds through a DSB intermediate, perhaps one generated by MutLγ [27]. A simple gap-filling model for the generation of expansions from a staggered DNA DSB arising during transcription or DNA repair has been suggested [27]. 

Very little is known about the factors that promote contractions. In the mouse model, factors that abolish expansions do not necessarily reduce contractions [24,25,28,30,42]. In fact, the frequency of contractions usually increases when the expansion frequency drops. This suggests that some, if not all, contractions occur via a mechanism that differs from the expansion mechanism and that when expansions are blocked, a process or processes that favors contractions predominates. This would be consistent with the observation that AGG interruptions, which are an important modifier of expansion risk, do not affect the contraction frequency [4]. Moreover, while transcription or open chromatin is required for expansion, contraction of methylated alleles can be seen in both mouse and humans, particularly in rapidly dividing cells such as those in the early embryo or in cells grown at low cell densities in vitro [10,17,53]. Contractions under these circumstances may reflect the difficulty the cell has in replicating the FX repeats [49,51,52], thus favoring cells in which repeats have been lost. Tandemly repeated sequences often contract via strand-slippage during replication in a variety of organisms [64,65,66]. Such a mechanism could explain the observed loss of AGG interruptions sometimes associated with contraction [4], if slippage occurred upstream of the interruption with re-priming of DNA synthesis occurring downstream of the interruption. Strand-slippage by the FX repeat also has the potential to generate point mutations within the repeat by frameshifting or limited intra-strand template switching with priming within the hairpin [67,68,69]. 

*Implications for humans:* Genetic factors that impact expansion may contribute to the increased expansion risk seen for PM carriers with a family history of FXS relative to carriers of similar PM alleles in the general population [70]. It may also account for why some individuals show more somatic expansion than others (Figure 3 and Figure 4). Interestingly, single nucleotide polymorphisms (SNPs) in genes including *ERCC6/CSB* [71], *MSH3* [21,72] and *FAN1* [19,20] are thought to modify disease risk in other Repeat Expansion Diseases via their effect on somatic expansion. A SNP in *MLH1*, the binding partner of *MLH3* in the complex MutLγ, has also been shown to have a similar effect [19]. Since most factors that affect expansion in somatic cells also affect expansion in germ cells in mice, it is likely that similar genetic factors would also impact the risk of intergenerational expansion. However, as illustrated by the differences between the effects of FAN1 and EXO1 mutations on expansion in different tissues in mice, some genetic factors may be more important modulators of expansion in some cells than in others and thus, may affect expansion differently in different organs. For example, a polymorphism in *FAN1* may result in increased expansion in brain but not necessarily in oocytes, while *EXO1* polymorphisms may result in increased expansion in gametes, but not liver or other somatic tissue. Thus, a thorough understanding of expansion predisposition may require testing of multiple tissues. 

## 7. The Frequency of Large Contractions and Expansions can be Underestimated

**Figure 5:**
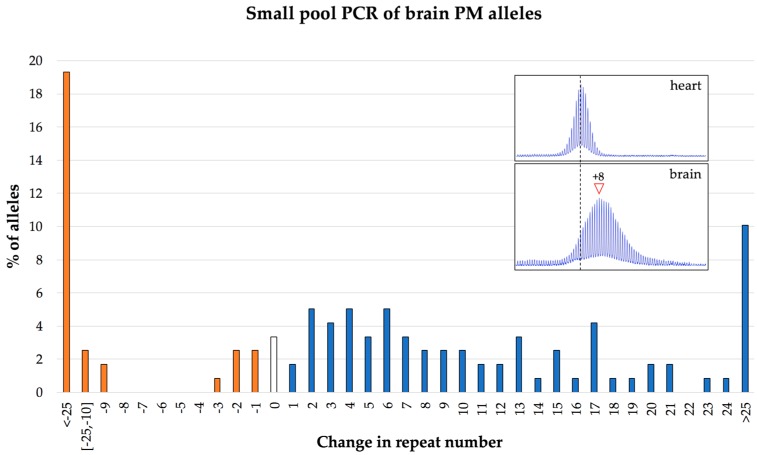
Distribution of the changes in the repeat number seen in the brain of a 1-year old mouse with an inherited allele of 162 repeats. The repeat number of individual alleles was determined by small pool PCR of single genome equivalents as described previously [30]. The data shown represent 119 individual PCR reactions. The inset panels show the bulk PCR profiles for the heart and brain of the same animal.

Analysis of repeat length and somatic instability is routinely performed on bulk genomic DNA. Such analysis on mice tissue indicates that somatic instability mostly involves the gain of relatively small number of repeats (Figure 2). However, large expansions and contractions can be seen in intergenerational transmission in mice [17]. They can also be seen if they occur during early embryonic development when they represent a significant fraction of the alleles in the population. These observations indicate that large expansions and contractions can occur not only in humans but also in mice. However, similar events occurring postnatally are difficult to detect using PCR on bulk genomic DNA since the resultant alleles vary considerably in the number of repeats gained or lost. Thus, each of these alleles represents a very small proportion of alleles in the population and likely will not be detected in standard PCR analysis. For example, as seen in Figure 5, when bulk DNA from the brain of a 1-year old mouse is analyzed, the PCR profile is consistent with most changes in repeat number involving the gain of a small number of repeats. However, PCR on single genome equivalents from the same brain sample shows that almost 30% of alleles have lost or gained more than 25 repeats. Thus, larger expansions and contractions actually occur relatively frequently and may ultimately reflect a relatively large fraction of the total alleles in the population. 

*Implications for humans:* This combination of expansion and contraction can result in individuals being highly mosaic for a variety of different alleles. The fact that larger expansions and contractions that occur later in development are difficult to detect in mice, raises the possibility that some humans may be even more mosaic than analysis of their bulk DNA suggests. Thus, careful analysis of the distribution of allele sizes in carriers might be needed to properly assess disease risk.

## 8. Concluding Remarks

The use of a mouse model allows the dynamics of repeat instability in the *FMR1* gene to be explored over time in multiple tissues. This has resulted in a number of observations that may be relevant to repeat instability in humans. For example, we have learnt that some cell types show more expansion than others [16,17,24,26]. In particular, expansions are more extensive in brain and gonads than in blood. This may mean that, in some people, the size of the repeat or the extent of somatic expansion seen in blood may not reflect what is present in disease-relevant cells. Examination of the repeat PCR profiles from mice over time has also shown that the σ of the allele peak provides a sensitive measure of the extent of somatic instability. Specifically, stable alleles have a low σ (<2.5), while expanded alleles have a larger σ. A wide range of σ values can be seen for human PM alleles in blood that, by analogy with FXD mice, likely reflects a wide variation in the extent of somatic expansion in different people. 

A number of genetic factors that promote or protect against expansions in the FXD mouse model have been identified [24,25,27,28,30,42,44,60,63,73,74]. Some of these factors have been implicated in expansion in humans with other Repeat Expansion Diseases [20,21,75,76,77], suggesting that they may be relevant for human carriers of unstable *FMR1* alleles as well. If so, the prediction would be that polymorphisms in these factors would be modifiers of both germ line and somatic expansion risk in FX families. As such, people who have elevated activities of protective factors or reduced activities of factors that promote expansion may show little, if any, somatic expansion. In contrast, those with elevated activities of expansion-promoting factors or reduced activities of protective factors may show more somatic expansion. Evidence from other Repeat Expansion Diseases suggests that somatic expansion contributes to differences in the age at onset and disease severity [20,21,75,76,77]. In a recent study, women who showed PM allele mosaicism reported more severe symptoms than women who were not mosaic [39], suggesting that somatic instability may exacerbate PM symptoms. However, additional studies are needed to fully understand the contribution of somatic expansion to disease pathology in PM carriers. In any event, an increased propensity for somatic expansion likely indicates an increased propensity for germ-line expansion and thus an increased risk of intergenerational transmission of larger alleles. Additional work is also needed to assess whether the same genetic factors that affect expansion risk in mice also modulate the risk of somatic and intergenerational expansion of human FXD alleles.
